# From New Endocrine Entities Requiring New Approaches to New Approaches Leading to New Endocrine Entities

**DOI:** 10.3390/diagnostics13030518

**Published:** 2023-01-31

**Authors:** Mara Carsote, Claudiu-Eduard Nistor, Nina Ionovici

**Affiliations:** 1 Department of Endocrinology, Faculty of Medicine, “Carol Davila” University of Medicine and Pharmacy & “C.I. Parhon” National Institute of Endocrinology, Aviatorilor Ave, 011683 Bucharest, Romania; 2 Department 4—Cardio-Thoracic Pathology, Thoracic Surgery II Discipline, Faculty of Medicine, “Carol Davila” University of Medicine and Pharmacy & Thoracic Surgery Department, “Dr. Carol Davila” Central Emergency University Military Hospital, 013058 Bucharest, Romania; 3 Department of Occupational Medicine, Faculty of Medicine, University of Medicine and Pharmacy of Craiova, 200349 Craiova, Romania

The dynamics pace of modern society is reflected by the medical community, the public health concerns, the quality of life, as well as the specific spectrum of various disorders. The health impact of these worldwide events is even more visible, starting with early 2020 with the COVID-19 infection spreading from China [1,2,3]. The field of endocrinology is no exception to this scenario. We highlight three major directions: the first one is that COVID-19-positive patients were initially not considered to be at particular risk for most of the endocrine disorders. Since then, numerous endocrine gland-associated COVID-19 entities were seen over the next three years and the virus virtually affected any gland to different degrees [4]. The consequences of new regulations and norms during the first waves of the COVID-19 pandemic delayed the diagnosis and the management of many endocrine and non-endocrine disorders with a severe impact on general health (as, for instance, delayed surgery for candidates with thyroid cancer), while practitioners used telemedicine as a surrogate of daily physical consultations [5,6]. In the meantime, the general progress in science included cutting-edge aspects of endocrinopathies underlying genetic testing, advanced imaging and surgical procedures, new biomarkers or innovative molecular biology technologies, and, also, the development of multidisciplinary teams dedicated to a particular condition [7,8].

The COVID-19 pandemic required new medical approaches due to its unexpected severity, multileveled social outcomes, and dramatic overall impact. Of the endocrine issues, the thyroid represents one of the most affected glands, either in terms of subacute thyroiditis due to hosting the virus at this level, a thyroid dysfunction accompanying severe illness, or following a pituitary damage or an abnormal autoimmunity amidst stress or infectious triggers. COVID-19-associated subacute thyroid inflammation also takes on a less severe form after COVID-19 vaccination, which requires awareness. This development has not affected the immunization process as far as we know [9,10,11,12].

Another major direct impact concerns the genetic background of unusual endocrine cases. The current availability and increased access to genetic testing, for example, in some conditions underlying a short stature or late onset congenital adrenal hyperplasia, might provide a better understanding of these hormonal anomalies, and thus potentially improve clinical practice [13,14,15]. Besides the special importance of genetic diagnosis in pediatric endocrinology, it is also useful in adults as, for instance, thyroid malignancies [16,17]. It is also particularly important in the genetic evaluations for various endocrine syndromes with autoimmune or tumor components such as multiple endocrine neoplasia [18,19,20].

Trans-disciplinary approaches involving teams of different specialists in addition to a specialist in endocrinology are important either for providing surveillance for a non-endocrine disorder with endocrine consequences or for severe endocrine pathologies with general effects on overall health (as seen in endocrine oncology, cardiovascular endocrinology, etc.) [21]. Clinicians of different medical domains are needed to follow females with beta-thalassemia, including during pregnancy (a rare event, but more common than a decade ago), due to advances in the hematological field and pre-conception counselling. Endocrine involvement in this challenging condition includes almost every gland and has been recently recognized as thalassemic endocrine disease [22,23]. In addition to the progress in gynecological endocrinology and the assisted reproduction techniques, we have more cases of pregnancy in hormonally active disorders as, for instance, in females with active acromegaly [24]. Dermato-endocrinology might represent another valuable inter-disciplinary area. Of note, most of the endocrine entities might have a skin and/or an oral clinical expression [25,26]. A case in point is the presence of acanthosis nigricans as a practical and informative tool of insulin resistance accompanying cardio-metabolic conditions with a high epidemiological impact as diabetes mellitus or metabolic syndrome [27,28]. Moreover, the progress of steroid metabolomics, and molecular genetics, includes some aspects of adrenal disorders, such as CYP11B1/B2 84 immunostaining providing more information on particular entities as Conn’s or Cushing’s syndrome, adrenal incidentalomas, or adrenal cortical carcinoma [29,30].

Integrating these new aspects of every day endocrine practice into a multidisciplinary constellation of approaches in addition to the recent global health concerns and social issues represent a step forward for the benefit of our patients.
